# Comprehensive Collection of Whole-Slide Images and Genomic Profiles for Patients with Bladder Cancer

**DOI:** 10.1038/s41597-024-03526-3

**Published:** 2024-06-27

**Authors:** Pei-Hang Xu, Tianqi Li, Fengmei Qu, Mingkang Tian, Jun Wang, Hualei Gan, Dingwei Ye, Fei Ren, Yijun Shen

**Affiliations:** 1 Department of Urology, Fudan University Shanghai Cancer Center, Shanghai, China; 2 Department of Oncology, Shanghai Medical College, Fudan University, Shanghai, China; 3 Department of Pathology, Fudan University Shanghai Cancer Center, Shanghai, China; 4 Institute of Pathology, Fudan University, Shanghai, 200032 China; 5 Jinfeng Laboratory, Chongqing, 401329 P.R. China; 6 Department of Urology, Sun Yat-sen University Cancer Center, Guangzhou, China; 7 State Key Laboratory of Oncology in Southern China, Guangzhou, China; 8 State Key Laboratory of Oncology in South China, Collaborative Innovation Center for Cancer Medicine, Sun Yat-sen University Cancer Center, Guangzhou, China; 9 State Key Lab of Processors, Institute of Computing Technology, CAS, Beijing, 100190 China

**Keywords:** Bladder cancer, Computational models

## Abstract

Bladder cancer is one of the leading causes of cancer-related mortality in the urinary system. Understanding genomic information is important in the treatment and prognosis of bladder cancer, but the current method used to identify mutations is time-consuming and labor-intensive. There are now many novel and convenient ways to predict cancerous genomics from pathological slides. However, the publicly available datasets are limited, especially for Asian populations. In this study, we developed a dataset consisting of 75 Asian cases of bladder cancers and 112 Whole-Slide Images with one to two images obtained for each patient. This dataset provides information on the most frequently and clinically significant mutated genes derived by whole-exome sequencing in these patients. This dataset will facilitate exploration and development of novel diagnostic and therapeutic technologies for bladder cancer.

## Background & Summary

Bladder cancer (BLCA) is one of the most common and fatal malignant tumors of the urinary system^1^. Bladder cancer genomics aims to understand the genetic basis of tumor cell proliferation and evolution of the cancer genome under mutation and selection by the environment within the body, as well as the immune system and therapeutic interventions^2–5^. In medicine, the main reasons for knowing the cumulative phenotypic consequences of somatically acquired genetic, genomic and epigenetic alterations in cancer cells are related to prevention, detection and treatment^6,7^. For example, the presence of TP53 and ERCC1 mutations might influence the sensitivity of muscle-Invasive bladder cancer (MIBC) to chemotherapy^8,9^. Furthermore, FGFR3 mutations characterize a subgroup of bladder cancers with a good prognosis, and EGFR, OAS1 and MST1R mutations indicate sensitivity to immunotherapy^10,11^. The ability to identify these genetic mutations has benefits in terms of treatment of bladder cancer.

Traditionally, acquisition of a cancerous genome requires tumor resection, quality control and sequencing or panel detection, which is time-consuming, labor-intensive and expensive^12^. Identification of the genomic features of a tumor has significance for treatment and the prognosis^13^. Numerous studies have attempted to predict the genomic status of tumors from pathological sections or even images^14^. However, training such models typically requires a large amount of data, and publicly available datasets are scarce^15^. Most of the current studies are based on The Cancer Imaging Archive (TCIA)^16^ data and lack publicly available external cohorts for validation, which has a negative impact on the robustness and extensibility of the models. Meanwhile, The Cancer Genome Atlas (TCGA) sequencing data focus predominantly on the Caucasian population, and further validation is needed to determine whether models trained on the TCGA database can be applied to other ethnic groups.

**Fig. 1:**
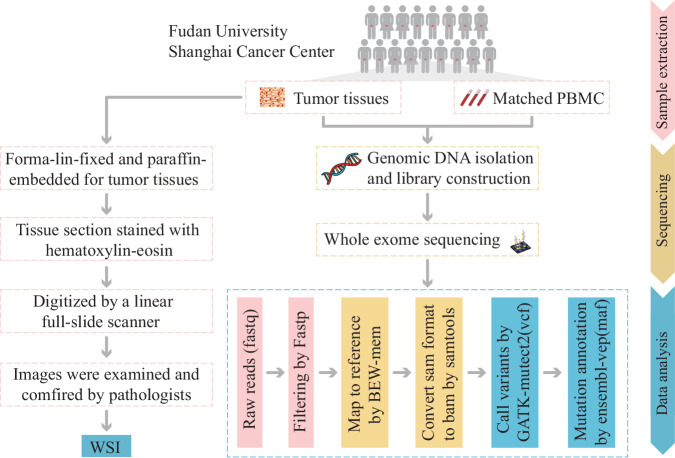
A schematic overview of this study design and workflow. PBMCs, peripheral blood mononuclear cells; WSI, whole-slide image.

We have previously published a large single-center artificial intelligence study in which the genomic features of 75 Asian patients with muscle-Invasive bladder cancer were successfully predicted by whole-slide images (WSIs)^17^. Tissues from these patients, all of whom had stage II or higher bladder cancer, were sequenced, sectioned with hematoxylin-eosin staining, and scanned for WSIs. To facilitate the reuse of these unique datasets, we described the datasets including WSIs and mutational information from whole-exome sequencing(WES) data for bladder cancer in detail with specific attention to data quality. A schematic overview of the study design and workflow is shown in Fig. 1.

## Methods

### Patients and ethical approval

The study was approved by the research ethics committee of the Fudan University Shanghai Cancer Center (FUSCC; approval number 2006218-Exp3). All specimens for 75 patients were obtained retrospectively from the histopathology archive with approval from the FUSCC. The requirement for informed consent was waived by the ethics committee. The clinical information for the 75 patients was shown in Table S1.

### Selection and preparation of specimens

Tumor tissues from the 75 patients were routinely fixed in formalin and embedded in paraffin. The tissue sections were stained with hematoxylin-eosin using an automated slide-staining machine (Autostainer XL + TS5015 + CV5030, Leica, Wetzlar, Germany). Case selection was randomized but only specimens with acceptable tissue quality were included. All images were digitized using a linear full-slide scanner (SQS600P, Teksqray, Shenzhen, China) at a resolution of 0.25 micrometers (40x) per pixel. All images were examined by two pathologists working independently and were found to show no evidence of any significant changes in intensity or color.

### Pathological assessment and quality control

We implemented a rigorous selection and quality control process to ensure the quality and consistency of pathological assessment. Histology slides were selected based on their quality and the presence of well-preserved tumor tissues. Slides showing significant artifacts and those with poor staining quality were excluded. Tumor areas in the whole-slide images (WSIs) were identified and marked by two pathologists working independently, and included based on the presence of viable tumor cells and minimal contamination by non-tumor tissue. Any discrepancies between the two pathologists were resolved by consensus. Each WSI was then reviewed for image clarity, color consistency, and accurate representation of the tumor area. If any quality issues were detected, the images were rescanned to ensure the highest quality data.

### Instrumentation

The WSIs of bladder cancer were acquired using a digital scanner (Leica AT Turbo, Leica) with a 40 × objective lens. The average dimensions of the WSIs were 97389 × 80638 pixels with a physical size of 24.16 × 19.94 mm.

### DNA preparation

Fresh tumor tissues and matched peripheral blood mononuclear cells (PBMCs) from 75 patients were obtained from the FUSCC tissue bank. DNA was extracted from the PBMCs using a DNA kit and placed in tubes containing EDTA as an anticoagulant. The tumor tissues were frozen at −80 °C in liquid nitrogen. The collected DNA was extracted again using a DNeasy Blood & Tissue Kit (Qiagen, Venlo, Netherlands).

### Whole exome sequencing

DNA staining was performed for tumor tissues and matched PBMCs using an E210 system (Covaris, Woburn, MA,USA), with 100 ng of DNA cut to obtain an average fragment size of 200 base pairs. Next-generation sequencing libraries for tumor gDNA matched to the gDNA for PBMCs were prepared using DNA library kits (Agilent v6, Agilent Technologies, Santa Clara, CA, USA). A fluorometer and a 2100 Bioanalyzer (Agilent Technologies) were used to measure the mass and fragment size of the prepared library. The libraries were loaded onto a Nextseq platform (Illumina, San Diego, CA, USA) for pair-end sequencing with a read length of 150 base pairs.

### Data processing and quality control of WES data

Fastp^18^ corrected the raw sequencing data by quality control, read filtering and base correction. The cleaned data were also aligned to the reference human genome (UCSC hg38) using the Burrows-Wheeler Aligner^19^. After removal of duplications and local rearrangements by SAMtools^20^, core single nucleotide variants and insertions and deletions were identified using Mutect2^21^. Next, somatic alterations were obtained after removing germline alterations from a matched blood sample. We fixed mate pairs, marked PCR duplicates and performed the base quality recalibration using a GATK4 Genome Analysis Toolkit^22^. Variants labeled as “PASS” were kept for downstream analysis. Variations were annotated using the Ensembl Variant Effect Predictor (VEP)^23^.

## Data Records

The TCIA datasets mentioned in the Background & Summary section can be accessed through TCIA (10.7937/K9/TCIA.2016.8LNG8XDR)^24^. The datasets for the 75 patients with bladder cancer have been recorded as 112 WSIs files, along with a summary containing mutational information for 16 genes from the WES data (Table S1). The mutational information for the 75 tumor tissues specimens is annotated by VEP containing only PASSed variants. All WSIs are provided as SVS files. Details of the raw sequencing data and matched WSIs are shown in Table S2. These files have been deposited in The National Omics Data Encyclopedia (NODE) (OEP004732)^25^. The raw sequencing of bladder cancer and germline data have been deposited in the National Genomics Data Center (NGDC) (HRA007156)^26^.

## Technical Validation

### Quality control and validation of WSIs

**Fig. 2:**
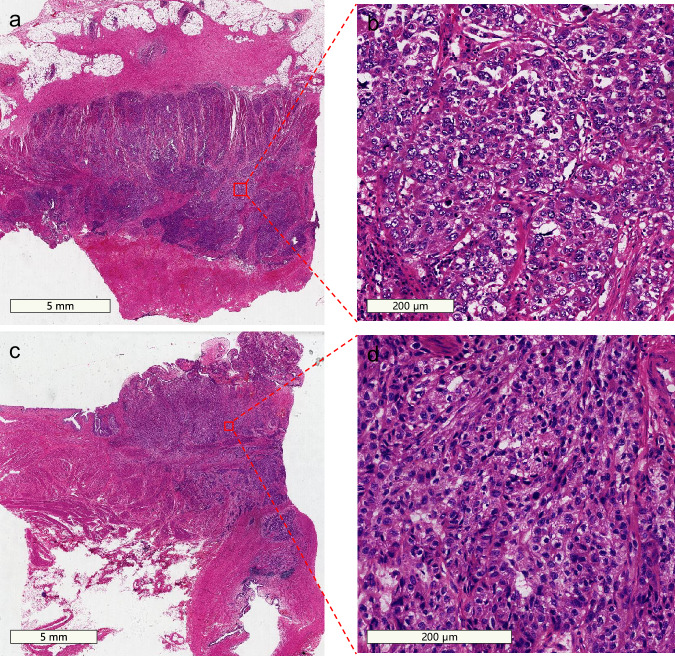
Whole-slide images of tumor tissues from two bladder cancer patients. (**a**) Whole-slide image of patient 1. (**b**) Magnified view of the tumor region from patient 1. (**c**) Whole-slide image of patient 2. (**d**) Magnified view of the tumor region from patient 2.

After successfully scanning each slide on the whole slide scanner, a pathologist first independently reviewed all cases in the form of digital slides, and each WSI was reviewed by two pathologists working independently. The pathologists reported all cases according to their routine workflow. The quality and consistency of the slide digitization process was primarily managed and regulated by the WSI quality control module, so as to improve the accuracy and reliability of the digital pathological diagnosis. The quality of scans, sections, diagnosis and data were mainly determined by the pathologists. Figure 2a,c and partially enlarged images (Fig. 2b,d) were independently confirmed to be WSIs of bladder cancer by the two pathologists.

**Fig. 3:**
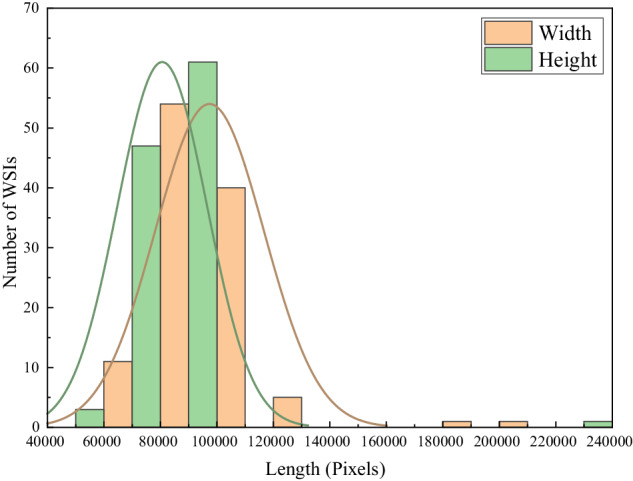
Distribution of width and height of whole-slide images in pixels. WSIs, whole-slide images.

Our dataset consisted of 112 WSIs from 75 patients. We counted the distribution of WSIs width and height of the WSIs in pixels. Figure 3 showed that the majority of WSIs had a width in the range of 80,000-90,000 pixels and a height in the range of 90,000-100,000 pixels.

### Quality control and evaluation of WES

**Fig. 4:**
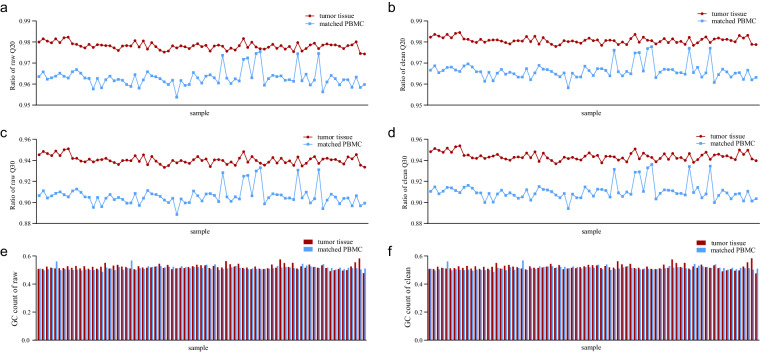
Quality control of sequencing data. (**a**–**d**) The Q20/Q30 of the raw and clean sequencing data. (**e,f**) The GC content of the raw and clean sequencing data. Each spot or bar on the x-axis represents a single sample. PBMCs, peripheral blood mononuclear cells.

We used Fastqc^27^ to assess the quality of the raw data. Fastp was then used to rectify the raw sequencing data by quality control, read filtering, and base correction. Figure 4 showed the Q20/Q30 line charts and the GC content for both the raw and clean data for the 75 samples. The Q20 value (indicating a 99% probability of correct identification) and Q30 value (indicating a 99.9% probability of correct identification) for the clean data surpassed 98% and 94%, respectively, for tumor tissues and 96% and 90% for PBMCs after quality control in most samples. The GC contents remained relatively stable, indicating that no significant errors were introduced during quality control and the consistency of the data was maintained. All data files fell within acceptable parameters.

**Fig. 5:**
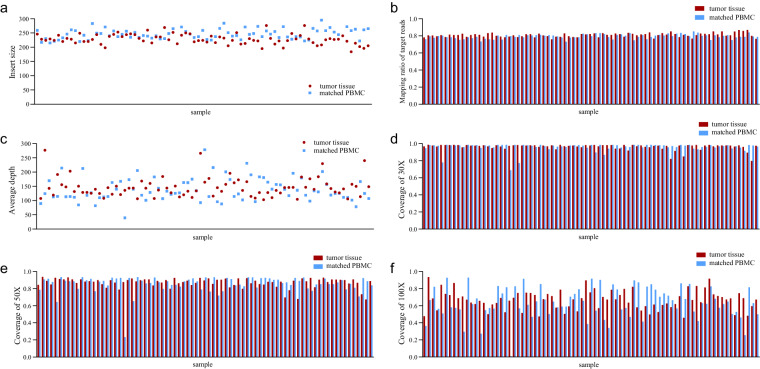
Confirmation of sequencing quality and coverage. (**a**) Insert size distribution of tumor tissues and matched PBMCs. (**b**) Mapping ratio of target reads for tumor tissues and matched PBMCs. (**c**) Average depth of coverage for tumor tissues and matched PBMCs. (**d**–**f**) Coverage at 30×, 50× and 100× for tumor tissues and matched PBMCs. Each spot or bar on the x-axis represents an individual sample. PBMCs, peripheral blood mononuclear cells.

Qualimap^28^ was used to analyze the sequencing alignment data and assess the features of the mapped reads to identify biases in the sequencing and mapping processes, thereby facilitating decision-making for further analysis. The insert size of bladder cancers and PBMCs were in the range of 184-326 base pairs, resulting in a more uniform read coverage for WES^29^ (Fig. 5a). The mapping ratio of target reads between tumor tissues and PBMCs was similar, with both hovering around 80% (Fig. 5b). The average depth was calculated to be 146.1 for tumor tissues and 138.6 for PBMCs (Fig. 5c). We also compared the genomic coverage between tumor tissues and PBMCs using coverage depths of 30×, 50×, and 100× as shown in Fig. 5d–f. All the aforementioned data were within the standard range.

### Statistics and evaluation of variation

**Fig. 6:**
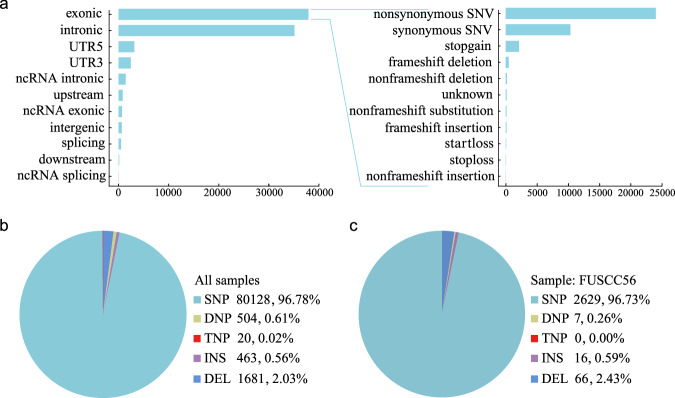
Annotation of identified variants. (**a**) Genomic location and the main variant consequences. Classification of variants for all samples (**b**) and a single sample (**c**). DEL, deletion; DNP, double nucleotide polymorphism; INS, insertion; SNP, single nucleotide polymorphism; SNV, single nucleotide variant; TNP, triple nucleotide polymorphism; UTR, untranslated region.

We aligned the sequences to the hg38 reference human genome and annotated them using VEP. The variants were predominantly categorized as exonic or intronic, with most exonic variants being nonsynonymous single nucleotide variants, synonymous single nucleotide variants and stop-gain mutations (Fig. 6a). The majority of variants were single nucleotide polymorphisms which accounted for 96.78% of case, while the rest were deletion, double nucleotide polymorphism, insertion, and triple nucleotide polymorphism variants (Fig. 6b). Figure 6c showed the variants observed in a single sample.

We analyzed and counted the top ten mutated genes, including TP53, KMT2D, and KDM6A, in 75 tumor tissues (Table S1). We also documented six other clinically significant genes (Table S1). ERCC2, ERBB2, ATM, and RB1 were associated with the effect of chemotherapy or immunotherapy^17^. FGFR3 was related to targeted therapy and the prognosis^30^, while PIK3CA could serve as a potential biomarker for targeted therapy^31^. Furthermore, we analyzed single nucleotide variants and copy-number variations for all genes. Detailed information on the variations was provided in Tables S3 and S4.

**Fig. 7:**
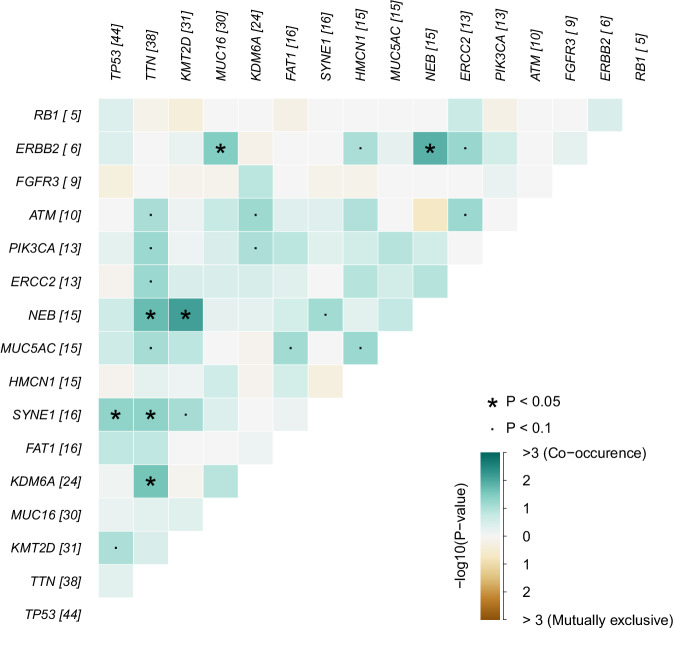
Heatmap for mutational associations between 16 genes. The color intensity reflects co-occurrence of mutations, with darker colors indicating stronger co-occurrence. Asterisks (*) denote p < 0.05, indicating a highly significant correlation, while a dot (·) represents 0.05 < p < 0.1, indicating a tendency toward significance.

A heatmap illustrating the associations between the mutations of these 16 genes was shown in Fig. 7. Each square in the figure represented the correlation between two genes, with the depth of color indicating the co-occurrence of mutations. Darker colors indicated stronger co-occurrence and lighter colors suggested that mutations in the two genes were more likely to be mutually exclusive. An asterisk indicated a statistically significant correlation, for example, NEB and KMT2D. A dot indicated a tendency toward significance, as with ERBB2 and ERCC2. The results showed that most of these mutations displayed co-occurrence (Fig. 7), indicating that these genes might work together in similar pathways, potentially contributing to the development and progression of bladder cancer.

## Usage Notes

The dataset for the WSI files described in this article can be downloaded from The National Omics Data Encyclopedia (https://www.biosino.org/node) by pasting the accession number OEP004732 into the text search box or from the URL: https://www.biosino.org/node/project/detail/OEP004732. The raw sequence data reported in this paper have been deposited in the Genome Sequence Archive (Genomics, Proteomics & Bioinformatics 2021) in National Genomics Data Center (Nucleic Acids Res 2022), China National Center for Bioinformation / Beijing Institute of Genomics, Chinese Academy of Sciences (GSA-Human: HRA007156) that are publicly accessible at https://ngdc.cncb.ac.cn/gsa-human.

The sequencing data were processed by freely available and open access tools. The sequencing data for the matched PBMCs was used to generate a panel of normal in the step of variant detection conducted by Mutect2, confirming that we only provided the mutational information for tumor tissues.

The WSIs can be viewed by ImageScope (https://www.leicabiosystems.com/digital-pathology/manage/aperio-imagescope/). The clinical and mutational information were shown in Table S1. The filenames of the WSIs and raw sequencing data are provided in Table S2. The single nucleotide variants and copy-number variation can be found in Table S3 and Table S4, respectively.

### Supplementary information

Table S1. Clinical information of 75 patients

Table S2. The details of WSIs and sequencing data

Table S3. The copy-number alterations of 75 patients

Table S4. The single nucleotide variants of 75 patients

## Data Availability

We used a typical workflow to process the WES data. All software used in this study is freely available: Fastp: https://github.com/OpenGene/fastp. BWA: https://github.com/lh3/bwa. GATK: https://github.com/broadinstitute/gatk/releases. VEP: https://m.ensembl.org/info/docs/tools/vep/script/vep_download.html. No code was used in the generation of the WSIs files. No code is required to access or analyze this dataset.
